# 24-h Movement Guideline Adherence and Mental Health in University Students: Patterns Across Adherence Levels and Academic Fields

**DOI:** 10.3390/bs16050766

**Published:** 2026-05-13

**Authors:** Laura García-Pérez, Gema Torres-Luque, Clarice Maria Lucena Martins, Rosario Padial-Ruz

**Affiliations:** 1 Department of Didactics of Musical, Visual Arts, and Corporal Expression, Faculty of Education, University of Granada, 18071 Granada, Spain; rpadial@ugr.es; 2 Department of Didactics of Musical, Visual Arts, and Corporal Expression, Faculty of Humanities and Education Sciences, University of Jaén, 23071 Jaén, Spain; 3 Research Center on Physical Activity, Health and Leisure, Faculty of Sport, University of Porto, 4200-450 Porto, Portugal; clarice@fade.up.pt

**Keywords:** 24-h movement guidelines, university student, physical activity, sedentary behaviour, sleep, mental health

## Abstract

Adherence to the 24-h movement guidelines (24-HMG), which integrate physical activity (PA), sedentary behaviour, and sleep, may be relevant to university students’ mental health, yet evidence in this population remains limited. This cross-sectional study aimed to estimate adherence to the 24-HMG, identify associated correlates, and examine whether meeting a greater number of guidelines was associated with more favourable mental health profiles in Spanish university students. A total of 1469 students (mean age = 21.6 ± 3.14 years; 71% women) completed validated self-report measures of physical activity, sedentary behaviour, sleep duration, psychological distress, self-esteem, and resilience. Adherence was defined according to the Canadian adult 24-HMG, and a global adherence index (0–3) was calculated. Concurrent adherence to all three guidelines was low and varied markedly across academic fields, ranging from 22% in Sport Sciences to 1.6% in Engineering and Architecture. Women showed lower odds of meeting the physical activity recommendation and of meeting all three guidelines. Greater adherence was associated with a lower likelihood of unfavourable mental health profiles, particularly low self-esteem and low resilience. These findings suggest that adherence to the 24-HMG was low in this sample of university students and support the development of integrated, context-tailored interventions targeting movement behaviours and mental health in university settings.

## 1. Introduction

Accumulating evidence indicates that key day-to-day behaviours—engaging in sufficient physical activity (PA), limiting sedentary time, and obtaining adequate sleep—are each associated with favourable health outcomes, including better cardiometabolic health, lower all-cause health risk, and more favourable psychological well-being (Kracht et al., 2024). Importantly, these behaviours are interdependent: daily time is finite (1440 min), and increasing time in one behaviour necessarily requires decreasing time in another (Mekary & Ding, 2019). This premise has informed the development of time-use epidemiology and analytical approaches such as compositional data analysis and isotemporal substitution models, which have advanced understanding of how reallocations of time between PA, sedentary behaviour, and sleep may relate to health outcomes (Dumuid et al., 2018). At the same time, this integrated perspective has also supported the development of practical guideline-based frameworks for public health research and surveillance. Within this context, the Canadian Society for Exercise Physiology released the first 24-h Movement Guidelines for adults (18–64 years) in 2020, formalising the concept of a “healthy day” by integrating recommendations for PA, sedentary behaviour, and sleep within a single framework (Ross et al., 2020).

University students represent a priority population in which to examine 24-h movement patterns. The transition to university is a period in which daily routines and health-related habits are often restructured due to greater autonomy in lifestyle decisions, elevated academic demands, routine disruption, and sustained screen exposure. Evidence suggests that this period is often accompanied by declines in PA, increases in sedentary time, and sleep disturbances, yielding a behavioural profile that may increase vulnerability to adverse physical and psychological health outcomes (Castro et al., 2018; Roberts et al., 2024). Accordingly, understanding how students allocate daily time across movement and rest is particularly relevant, as relatively small shifts during this life stage may help shape health trajectories over time.

An increasing body of evidence suggests that concurrent adherence to 24-h movement recommendations is associated with better mental health and well-being. Recent evidence indicates that higher adherence to the 24-h movement guidelines (24-HMG) tends to co-occur with more favourable psychological profiles and lower distress (Luo et al., 2025). In line with this broader time-use perspective, university-based studies using compositional and isotemporal approaches suggest that relatively small reallocations of time away from sedentary behaviour toward PA or sleep may be associated with more favourable outcomes such as lower perceived stress (Dong et al., 2025). Nevertheless, prior work has more frequently focused on risk-oriented outcomes (e.g., depressive symptoms and psychological distress), with comparatively less attention devoted to positive psychological resources—such as resilience and self-esteem—within an integrated 24-h framework, particularly in university samples. These constructs are theoretically relevant because they reflect adaptive psychological functioning, coping capacity, and perceived self-worth, which are central to students’ well-being and adjustment during the university period. These indicators are especially relevant in university populations because they are closely linked to coping with academic demands, psychological adjustment, and persistence in challenging educational environments (García-Pérez et al., 2025a, 2025b).

Despite its relevance, simultaneous adherence to all three guidelines remains low internationally. For example, approximately 9.9% of Canadian university students met all assessed components in the initial deployment of the Canadian Campus Wellbeing Survey (Weatherson et al., 2021), whereas higher prevalence has been reported in Chinese university samples (Zhang et al., 2024). This variability suggests that adherence is likely shaped by contextual, cultural, and methodological factors (e.g., operational definitions and measurement approaches). In university settings, academic fields may influence daily movement opportunities, curricular demands, screen exposure, and time pressure, whereas socioeconomic status (SES) may affect access to facilities, transport, material resources, and living conditions that facilitate or constrain both PA and sleep routines. These factors may influence regular physical activity by shaping the time, resources, and environmental opportunities available for active behaviours within everyday university life. However, evidence from Spain remains limited, and few studies have examined 24-HMG adherence in university students while explicitly considering these determinants.

Against this background, Spain-specific evidence is needed to inform targeted, context-sensitive approaches to improving 24-HMG adherence and related psychological outcomes in higher education. Accordingly, the primary aim of this study was to estimate the prevalence of 24-HMG adherence and to identify its sociodemographic and academic correlates among Spanish university students. The secondary aim was to examine whether the number of guidelines met (0–3) was associated with three mental health indicators—psychological distress, self-esteem, and resilience—thereby providing actionable evidence to support integrated, 24-h approaches to student well-being.

## 2. Materials and Methods

### 2.1. Study Design and Participants

We conducted an analytical cross-sectional study using an online self-report survey (non-experimental design). The target population comprised undergraduate and postgraduate students enrolled at a higher education institution in southeast Spain during the study period. Participants were recruited through voluntary, non-probability sampling by disseminating an institutional invitation to approximately 46,000 students via official university channels.

A total of 1652 responses were received. After applying exclusion criteria (questionnaires with essential information missing or internally inconsistent), 183 records were removed. The final analytic sample included 1469 students (mean age = 21.6 years, SD = 3.14), with 71% women and 29% men, and complete data on 24-h movement behaviours.

### 2.2. Procedure and Ethical Considerations

Data were collected between November 2024 and March 2025 through an online questionnaire distributed via institutional email and social media. Before participation, students received study information and provided informed consent electronically. Completion time was approximately 10 min, and participants were entered into a draw for a gift card redeemable for school or technology-related materials. The study was conducted in accordance with the Declaration of Helsinki and was approved by the Human Research Ethics Committee of the University of Granada (Protocol code: 3678/CEIH/2023, Approval Date: 26 September 2023). Data processing complied with the General Data Protection Regulation (EU 2016/679) and applicable Spanish regulations.

### 2.3. Measures

#### 2.3.1. Sociodemographic Covariates

Participants completed an ad hoc questionnaire assessing sociodemographic and lifestyle characteristics, including sex, age, and socioeconomic status (SES). SES was assessed via self-reported monthly household income and categorised into three levels: low (up to the minimum wage), medium (>minimum wage and <€3000/month), and high (≥€3000/month). These covariates were included in all adjusted models.

#### 2.3.2. 24-h Movement Behaviours and 24-HMG Adherence

**Physical activity (PA).** PA was assessed using the short form of the International Physical Activity Questionnaire (IPAQ-SF) (Craig et al., 2003). Internal consistency was acceptable (α = 0.792). Weekly energy expenditure (MET-min/week) was calculated following standard scoring procedures (walking = 3.3 METs; moderate PA = 4.0 METs; vigorous PA = 8.0 METs).

**Sedentary behaviour.** Sedentary behaviour was measured using the Youth Leisure Sedentary Behaviour Questionnaire (YLSBQ) applying the university-student adaptation proposed by Atencio-Osorio et al. (2021). Internal consistency was excellent (α = 0.872). Participants reported average time spent in sedentary activities over the past week, separately for weekdays and weekend days, using response categories (none, 30 min, 1 h, 2 h, 3 h, 4 h, ≥5 h). Categories were converted to minutes using midpoints (assigning 300 min to “≥5 h”). Average daily SB was computed as [((weekday × 5) + (weekend × 2))/7] and analysed in minutes/day.

**Sleep duration.** Sleep duration was obtained from the Spanish version of the Pittsburgh Sleep Quality Index (PSQI) (Macías-Fernández & Royuela-Rico, 1996), using the sleep-duration component (item 3: hours of actual sleep per night during the past month). Hours were converted to minutes/day by multiplying by 60. Internal consistency of the full PSQI was excellent (α = 0.890).

**Classification of adherence to the 24-HMG.** Participants were classified as meeting/not meeting each recommendation according to the Canadian 24-h Movement Guidelines for Adults (Ross et al., 2020; Zhang et al., 2024): (i) sleep duration of 7–9 h/night; (ii) sedentary behaviour ≤ 8 h/day; and (iii) PA ≥ 600 MET-min/week. A global adherence index (0–3) was created by summing the number of recommendations met. This guideline-based classification was used to align the analyses with the 24-HMG framework and to facilitate interpretation from a public health perspective.

#### 2.3.3. Mental Health Indicators

**Psychological distress.** Distress was assessed using the 21-item Depression Anxiety Stress Scales (DASS-21), validated in Spanish populations (Daza et al., 2002). Internal consistency was excellent (α = 0.950). Subscale scores (depression, anxiety, stress) were calculated by summing corresponding items; higher scores indicate greater distress. For the present analyses, a global DASS-21 score was used to capture overall psychological distress as a parsimonious indicator of unfavourable emotional symptomatology.

**Self-esteem.** Self-esteem was measured using the Rosenberg Self-Esteem Scale (Rosenberg, 2015), adapted for Spanish populations by Atienza et al. (2000). Reliability was high (α = 0.861). The instrument includes 10 items (five reverse-coded: items 2, 5, 6, 8, and 9) rated on a 4-point Likert scale. Total scores range from 10 to 40, with higher scores indicating higher self-esteem.

**Resilience.** Resilience was assessed using the 10-item Connor–Davidson Resilience Scale (CD-RISC-10) (Connor & Davidson, 2003), validated in Spanish young adults (Notario-Pacheco et al., 2011). Reliability was high (α = 0.859). Items are rated from 0 to 4, yielding total scores from 0 to 40; higher scores indicate greater resilience.

For logistic regression analyses, mental health outcomes were dichotomised to identify relatively unfavourable profiles within the sample. Psychological distress was classified as high when the global DASS-21 score fell in the upper quartile (≥P75). Self-esteem (Rosenberg; 10–40) was classified as low using a cut-off of <25, in accordance with commonly used score ranges, and resilience (CD-RISC-10; 0–40) was classified as low when scores fell in the lower quartile (≤P25). These thresholds were used for analytic interpretability and should not be interpreted as clinical diagnostic cut-offs.

### 2.4. Statistical Analysis

Descriptive statistics are presented as frequencies and percentages. Differences in the distribution of sociodemographic and academic variables across levels of global 24-HMG adherence (0–3 guidelines met) were examined using Pearson’s χ^2^ test. When contingency tables contained low expected cell counts, simulated *p*-values based on 10,000 Monte Carlo replicates were reported to improve robustness.

To identify correlates of meeting each guideline (physical activity, sedentary behaviour, and sleep) and of meeting all three guidelines, binary logistic regression models were fitted, and results are reported as adjusted odds ratios (AORs) with 95% confidence intervals (95% CIs). Models were adjusted for sex, age, SES, and academic field. Reference categories were men, 22–30 years, low SES, and Sport Sciences. Sport Sciences was selected as the reference category because it represented the field with the highest expected exposure to movement-related training and the highest observed prevalence of adherence, thus providing a theoretically meaningful comparison point. Age was grouped into 18–19, 20–21, and 22–30 years to reflect the sample distribution and to distinguish younger undergraduate students from an older group that included later-year undergraduate and postgraduate students.

To examine associations between global 24-HMG adherence (0–3 guidelines met) and mental health outcomes, logistic regression models were fitted and reported as crude odds ratios (CORs) and AORs with 95% CIs. The reference group was students meeting 0 guidelines, with comparisons for meeting 1, 2, and 3 guidelines.

Statistical significance was set at *p* < 0.05. Analyses were conducted using IBM SPSS Statistics (version 27.0) and R (version 4.5.1).

## 3. Results

### 3.1. Sample Characteristics and Adherence to the 24-HMG

The final sample comprised 1469 university students (mean age = 21.6 years, SD = 3.14), with women representing 71% of participants. As shown in Table 1, global 24-HMG adherence (0–3 recommendations met) differed significantly by sex (*p* = 0.005) and academic field (*p* < 0.001). Men were overrepresented in the highest adherence category (three guidelines met), whereas women were more prevalent in the lower adherence categories. Academic field emerged as a key differentiator: Sport Sciences students showed the highest prevalence of meeting all three guidelines (22%), whereas Arts and Humanities and Engineering and Architecture showed substantially lower prevalence (4.2% and 1.6%, respectively). Global adherence did not differ significantly by age group or socioeconomic status.

Multivariable logistic regression models identified distinct correlates of meeting each 24-HMG component and of meeting all three recommendations (Table 2). Compared with men, women had lower odds of meeting the PA recommendation (AOR = 0.44, 95% CI 0.33–0.59) and of meeting all three recommendations (AOR = 0.71, 95% CI 0.53–0.96). SES was positively associated with meeting the PA recommendation; students with high SES had greater odds of meeting PA than those with low SES (AOR = 1.94, 95% CI 1.03–3.76).

### 3.2. Correlates of Guideline Adherence

The academic field showed the largest differences in adherence. Using Sport Sciences as the reference category, all other fields had substantially lower odds of meeting the PA recommendation (AORs ≈ 0.05–0.12) and lower odds of meeting all three recommendations. For example, Engineering and Architecture students exhibited the lowest odds of global adherence (AOR = 0.20, 95% CI 0.07–0.48). Age was associated with sleep: students aged 18–19 years were less likely to meet the sleep recommendation than those aged 22–30 years (AOR = 0.62, 95% CI 0.46–0.84).

### 3.3. Global Adherence (0–3 Guidelines Met) and Mental Health Indicators

Table 3 summarises the associations between global 24-HMG adherence (0–3 recommendations met) and mental health indicators. A consistent pattern was observed whereby higher adherence categories were associated with a lower likelihood of unfavourable psychological profiles after adjustment for sex, age, SES, and academic field. Relative to students meeting 0 guidelines (reference), meeting two or three guidelines was associated with lower odds of high psychological distress (AOR = 0.59 and 0.55, respectively). Associations were strongest for positive psychological resources: meeting all three guidelines was associated with substantially lower odds of low self-esteem (AOR = 0.40, 95% CI 0.23–0.71) and low resilience (AOR = 0.41, 95% CI 0.23–0.74).

## 4. Discussion

This study provides, to our knowledge, the first evidence in Spanish university students on the prevalence of 24-HMG adherence, its sociodemographic and academic correlates, and its associations with three psychological indicators. Overall, adherence to the integrated 24-HMG was low, with marked differences across academic fields. In addition, students meeting a greater number of recommendations tended to show lower odds of unfavourable psychological profiles, particularly for self-esteem and resilience.

With respect to prevalence, our findings reinforce that simultaneous adherence to the 24-HMG among university students is generally modest and context-dependent (Pengpid & Peltzer, 2025; Weatherson et al., 2021; Zhang et al., 2024). A distinctive contribution of the present study is the pronounced within-country heterogeneity by academic field. Sport Sciences students showed substantially higher concurrent adherence than students in fields such as Engineering and Architecture, supporting an “academic-field effect” whereby the training environment—through faculty culture, curricular exposure to movement, and the availability of practice opportunities—may shape daily movement patterns. This contextual influence may also operate alongside self-selection processes, whereby students with stronger exercise-related identity or affinity preferentially enrol in sport-related degrees (Spittle et al., 2021). Together, these mechanisms may help explain why non-sport fields consistently exhibit lower odds of meeting PA recommendations (Esposito et al., 2024; García-Pérez et al., 2023) and, consequently, lower global adherence. In technologically oriented degree programmes, high academic workload, prolonged sedentary behaviour, and performance-related stress may create additional barriers to healthy movement behaviours (Popescu et al., 2025). In addition, evidence in university students suggests that physical exercise is associated with sleep quality, further highlighting the importance of considering these behaviours within an integrated framework (Li et al., 2025).

Across countries, reported 24-HMG adherence spans a wide range, from lower estimates in Canada (≈9.9%) to higher prevalence in China (≈27.8–31.7%) (Weatherson et al., 2021; Zhang et al., 2024). This dispersion likely reflects both contextual influences (e.g., cultural and environmental factors) and methodological features—particularly differences in operational definitions and the use of self-report versus device-based assessment—which can materially shift adherence estimates across studies and settings.

When examining determinants of adherence, sex emerged as a salient correlate of meeting the PA recommendation and, consequently, of global 24-HMG adherence. In our sample, women were less likely than men to meet the PA guideline and to meet all three recommendations concurrently, consistent with evidence in university populations (Martínez-Sánchez et al., 2024) and broader international findings (Pengpid & Peltzer, 2025). Although our study did not directly assess explanatory factors, prior literature suggests that this disparity may be related to differences in the volume and intensity distribution of activity—particularly vigorous and muscle-strengthening activity—as well as other contextual influences on participation. In some university settings, previous research has identified several barriers that may help contextualise these disparities, including lower perceived support, safety concerns, body image-related worries, experiences of harassment, and non-inclusive environments (Espada et al., 2023; Prieto-González & Alkouatli, 2025). These factors were not assessed in the present study and should therefore be interpreted as possible contextual explanations rather than study-specific findings. Accordingly, our results underscore the importance of gender-responsive interventions aimed at reducing contextual barriers (e.g., safety concerns and harassment), strengthening social support, and expanding inclusive and accessible opportunities aligned with students’ preferences and motivations (Brown et al., 2024). In practice, such interventions could include women-only or beginner-friendly activity sessions, peer-support programmes, safer and more inclusive activity spaces, and communication strategies designed to reduce body image-related concerns and improve perceived belonging.

SES was also associated with adherence, primarily through PA. Consistent with prior work indicating that greater resources facilitate access to facilities, organised activities, and the logistical conditions needed to sustain routines (e.g., time, transport, and equipment) (Sastra et al., 2024), medium and high SES in our sample were linked to higher odds of meeting the PA recommendation. However, this advantage did not translate into higher concurrent adherence across all three behaviours. Sleep and sedentary behaviour appear to be shaped by additional drivers (e.g., daily workload, stress, and digital habits), and evidence suggests that sociodemographic correlates of meeting all 24-h movement recommendations concurrently are heterogeneous across studies and populations (Ferrari et al., 2022; Liangruenrom et al., 2020). In addition, sleep health inequalities are not explained by economic resources alone; behavioural and psychosocial mediators contribute substantially to sleep disparities (Etindele et al., 2022; Papadopoulos & Etindele, 2023). Overall, these findings support interventions that target the full day—PA, sedentary behaviour, and sleep—rather than focusing exclusively on exercise promotion, consistent with the integrated premise of the 24-HMG (Rollo et al., 2020; Ross et al., 2020). Beyond describing adherence patterns, a key contribution of this study is showing that adherence to the 24-HMG was associated not only with lower odds of psychological distress but also with more favourable positive psychological profiles, particularly self-esteem and resilience, in the university setting. Our data indicate that higher 24-HMG adherence categories were generally associated with lower odds of unfavourable mental health profiles. This pattern was consistent for self-esteem and resilience; notably, meeting all three guidelines was associated with approximately 60% lower odds of low self-esteem and 59% lower odds of low resilience.

Taken together, these correlates help identify priority groups for implementation; the next question is whether higher 24-HMG adherence translates into meaningful differences in mental health indicators in this population. This interpretation is consistent with evidence in young adults and university samples showing that concurrent adherence to adequate PA, lower sedentary time, and sufficient sleep is associated with more favourable mental health and well-being (Porter et al., 2023). Complementing this evidence, a growing body of research indicates that students who concurrently meet recommendations for physical activity, sedentary behaviour, and sleep tend to report better mental health and well-being (Dong et al., 2025; García-Pérez et al., 2023). Together, these findings support the rationale for integrated interventions targeting the full 24-h day (sleep–sedentary behaviour–PA), rather than siloed approaches focused on a single component.

A possible interpretation is that PA, sedentary behaviour, and sleep may relate to mental health through partially overlapping and complementary pathways. Although the 24-h framework is grounded in the finite nature of daily time, these behaviours should not be understood as operating through a simple one-to-one trade-off only. Their relationships are complex and may also reflect shared underlying determinants, such as stress, academic workload, living conditions, digital habits, and individual characteristics. Prior literature suggests several mechanisms that may help explain these associations. For example, PA has been linked to mood and coping-related outcomes through neurobiological, psychophysiological, and psychosocial pathways, including self-efficacy and social support (Hossain et al., 2024; Ren & Xiao, 2023). PA—particularly at moderate-to-vigorous intensity—has also been associated with more favourable sleep outcomes, while sedentary behaviour, especially screen-based sedentary time, has been associated with poorer mental health in university populations (Barbosa et al., 2024; Gao et al., 2023; Vestergaard et al., 2024). In addition, night-time screen exposure has been linked to difficulties in sleep initiation and maintenance, plausibly through disruption of circadian signalling, which may in turn contribute to greater psychological distress and poorer psychological functioning. However, these mechanisms were not directly examined in the present study and should therefore be interpreted as possible explanations derived from the broader literature rather than as conclusions supported directly by our data.

Overall, these considerations may help contextualise why more favourable profiles across movement, sedentary behaviour, and sleep could be associated with better psychological functioning, including self-esteem and resilience. From an applied perspective, our findings are consistent with the value of integrated university-based approaches addressing sleep, sedentary behaviour, and physical activity together, rather than fragmented programmes focused on a single component. In this context, ‘academic adaptation’ refers to students’ capacity to adjust to academic demands, daily routines, and the broader challenges of university life.

### 4.1. Strengths and Limitations

These findings should be interpreted in light of several limitations. First, the cross-sectional design precludes causal inference and prevents conclusions about directionality. Reverse causation cannot be ruled out; it is equally plausible that students with more favourable mental health profiles are more likely to engage in healthier movement behaviours and to meet a greater number of guidelines. Second, movement behaviours were assessed via self-report, which may introduce recall and social desirability bias and may overestimate adherence compared with device-based assessment. In particular, the IPAQ-SF is known to overestimate physical activity relative to objective methods, which may have affected the classification of adherence to the physical activity guideline and, consequently, the global 24-HMG adherence index. In addition, we did not assess or control for sleep-related disorders or other clinical conditions that may have influenced sleep duration and sleep-related behaviours. The use of cut-points and the dichotomisation of behavioural and mental health variables may also entail substantial information loss, reduce sensitivity to variability within categories, and introduce possible misclassification, particularly for individuals near the thresholds. Moreover, the use of sample-based quartiles for psychological distress and resilience may limit comparability with studies using normative or clinically anchored cut-offs. No sensitivity analyses using alternative cut-points were conducted; therefore, the robustness of the threshold-based classifications should be interpreted cautiously. Although the academic field was included as an adjustment variable to account for contextual heterogeneity in the sample, it may also represent a structurally relevant determinant of movement behaviour patterns. If so, adjustment for academic field may have attenuated the estimated total associations between 24-HMG adherence and mental health outcomes. Finally, non-probability sampling—together with the overrepresentation of women and students from some academic fields, particularly Education Sciences—limits representativeness and generalisability and may introduce self-selection bias. Accordingly, prevalence estimates and between-field differences should be interpreted with caution, as they may not fully reflect the distribution of movement behaviours in the wider university population.

Despite these limitations, the study has notable strengths. The large sample (n = 1469) supports robust subgroup comparisons. The integrated 24-h framework provides a practical public health perspective for jointly considering PA, sedentary behaviour, and sleep, although our operationalisation was based on simplified categorical adherence indicators rather than more comprehensive time-use analytic approaches. Finally, multivariable models adjusted for key covariates (sex, age, SES, and academic field) helped account for relevant sample heterogeneity and identify priority targets for intervention. Future research would benefit from complementary analyses using continuous indicators and more comprehensive time-use approaches.

### 4.2. Practical Implications

From an applied perspective, the findings support using the 24-HMG as an operational framework for university health promotion, moving beyond strategies focused solely on “doing more exercise.” Universities may benefit from multi-component programming that addresses the full day by combining strategies targeting physical activity, sedentary behaviour, and sleep. This could include: (a) increasing PA through low-barrier, flexible, and non-competitive opportunities, such as short 10–15 min activity sessions between classes and drop-in formats requiring no prior registration; (b) reducing sedentary time—particularly screen-based sedentary behaviour—through active breaks during teaching, movement-supportive learning environments, and digital self-regulation initiatives; and (c) strengthening sleep hygiene via brief, skills-based education targeting schedule regularity, night-time screen exposure, and academic stress management. This multibehaviour focus is consistent with the overall patterns observed, whereby meeting a greater number of components relates to more favourable psychological profiles.

Given the marked between-field differences, implementation should be adapted to the characteristics of different academic fields rather than applied uniformly across the university. Faculties with the lowest adherence (e.g., Engineering and Architecture; Arts and Humanities) may require tailored initiatives embedded in their academic context, such as brief in-class micro-interventions (e.g., 3–5 min movement breaks during lectures), realistic PA challenges adapted to student schedules (e.g., step-based or weekly activity goals), partnerships with campus sport services to facilitate referral and access to existing programmes, well-being tutoring, and short health literacy modules. Targeting 24-h habits may be particularly relevant in these fields due to lower adherence and potentially greater exposure to academically driven sedentary time and performance pressure.

At the institutional level, sustainability and scalability are more likely if these actions are embedded within a “health in all university policies” approach, coordinated across sport services, student health/psychology units, teaching staff, and university governance. Ongoing evaluation using pragmatic indicators (e.g., reach/participation, adherence to each 24-HMG component, and changes in mental health indicators) would support iterative refinement and longer-term implementation.

## 5. Conclusions

Our findings suggest that concurrent adherence to the 24-HMG was low in this sample of Spanish university students and varied markedly across academic fields. This pattern further suggests that the training environment—through social norms, academic workload, and opportunities for practice—may act as a structural determinant of daily movement behaviours. Accordingly, campus-wide “one-size-fits-all” policies may be insufficient unless they are adapted to the distinct constraints and opportunities of different academic contexts.

In addition, the results indicate that meeting a greater number of guidelines was associated with lower odds of unfavourable psychological profiles, particularly low self-esteem and low resilience, even after adjustment for key sociodemographic and academic covariates. These findings support the value of integrated approaches that address PA, sedentary behaviour, and sleep together, rather than focusing on a single behaviour in isolation.

From an applied perspective, the results support embedding the 24-HMG framework within university health promotion strategies and prioritising multi-component, context-tailored actions to reduce academic and screen-based sedentary time, strengthen sleep hygiene, and expand accessible and sustainable opportunities for physical activity. Given the cross-sectional design, longitudinal studies and intervention trials are needed to clarify directionality and better examine how changes in daily time-use composition relate to students’ mental health over time.

## Figures and Tables

**Table 1:** Sample and 24-h movement guidelines (HMG) levels.

		24-HMG Levels				
Variable	Total n (%) ^1^	0HMG ^1^	1HMG ^1^	2HMG ^1^	3HMG ^1^	*p*-Value ^2^
**Age**						0.800
18–19	279 (19%)	14 (19%)	77 (20%)	113 (17%)	64 (21%)	
20–21	544 (37%)	24 (33%)	144 (38%)	241 (37%)	113 (37%)	
22–30	646 (44%)	34 (47%)	163 (42%)	297 (46%)	129 (42%)	
**Sex**						0.005
Male	432 (29%)	18 (22%)	100 (25%)	202 (30%)	112 (36%)	
Female	1037 (71%)	63 (78%)	307 (75%)	466 (70%)	201 (64%)	
**Socioeconomic status**						0.074
Low	161 (11%)	15 (19%)	56 (14%)	61 (9.1%)	29 (9.3%)	
Medium	1232 (84%)	62 (77%)	332 (82%)	572 (86%)	266 (85%)	
High	76 (5.2%)	4 (4.9%)	19 (4.7%)	35 (5.2%)	18 (5.8%)	
**Degree field**						<0.001
Sport Sciences	187 (13%)	1 (1.2%)	26 (6.4%)	92 (14%)	68 (22%)	
Education Sciences	741 (50%)	28 (35%)	202 (50%)	353 (53%)	158 (50%)	
Social and Legal Sciences	122 (8.3%)	9 (11%)	40 (9.8%)	48 (7.2%)	25 (8.0%)	
Engineering and Architecture	54 (3.7%)	4 (4.9%)	25 (6.1%)	20 (3.0%)	5 (1.6%)	
Arts and Humanities	114 (7.8%)	15 (19%)	37 (9.1%)	49 (7.3%)	13 (4.2%)	
Health Sciences	129 (8.8%)	18 (22%)	36 (8.8%)	50 (7.5%)	25 (8.0%)	
Sciences	122 (8.3%)	6 (7.4%)	41 (10%)	56 (8.4%)	19 (6.1%)	

Note: ^1^ n (%); ^2^ Pearson’s Chi-squared test with simulated *p*-value (based on 10,000 replicates).

**Table 2:** Correlates of adherence to 24-h movement guidelines, N = 1469.

		Physical Activity		Sedentary Behaviour		Sleep		All 3 Guidelines	
	Category	AOR (95% CI)	*p*	AOR (95% CI)	*p*	AOR (95% CI)	*p*	AOR (95% CI)	*p*
Sex	Male	1 (Reference)		1 (Reference)		1 (Reference)		1 (Reference)	
	Female	0.44 (0.33 to 0.59)	<0.001	0.90 (0.70 to 1.15)	0.385	1.21 (0.93 to 1.57)	0.154	0.71 (0.53 to 0.96)	0.023
Age	22–30	1 (Reference)		1 (Reference)		1 (Reference)		1 (Reference)	
	18–19	0.94 (0.67 to 1.31)	0.718	1.15 (0.85 to 1.56)	0.357	0.62 (0.46 to 0.84)	0.002	1.01 (0.71 to 1.45)	0.937
	20–21	0.85 (0.65 to 1.11)	0.229	0.83 (0.65 to 1.06)	0.142	1.16 (0.90 to 1.50)	0.260	0.97 (0.72 to 1.31)	0.858
SES	Low	1 (Reference)		1 (Reference)		1 (Reference)		1 (Reference)	
	Medium	1.51 (1.04 to 2.19)	0.031	1.16 (0.81 to 1.65)	0.414	1.28 (0.89 to 1.84)	0.176	1.13 (0.73 to 1.80)	0.599
	High	1.94 (1.03 to 3.76)	0.044	1.34 (0.75 to 2.40)	0.33	1.11 (0.62 to 2.02)	0.725	1.26 (0.62 to 2.50)	0.514
Degree field	Sport Sciences	1 (Reference)		1 (Reference)		1 (Reference)		1 (Reference)	
	Education Sciences	0.12 (0.05 to 0.22)	<0.001	1.52 (1.08 to 2.15)	0.017	0.52 (0.35 to 0.76)	<0.001	0.55 (0.38 to 0.81)	0.001
	Social and Legal Sciences	0.08 (0.03 to 0.16)	<0.001	1.00 (0.61 to 1.63)	0.999	0.55 (0.33 to 0.94)	0.027	0.46 (0.26 to 0.81)	0.008
	Engineering and Architecture	0.09 (0.04 to 0.21)	<0.001	0.61 (0.32 to 1.15)	0.132	0.35 (0.18 to 0.68)	0.001	0.20 (0.07 to 0.48)	0.001
	Arts and Humanities	0.05 (0.02 to 0.11)	<0.001	0.82 (0.49 to 1.34)	0.423	0.50 (0.29 to 0.85)	0.0109	0.26 (0.12 to 0.50)	<0.001
	Health Sciences	0.09 (0.04 to 0.19)	<0.001	1.26 (0.78 to 2.04)	0.348	0.34 (0.21 to 0.57)	<0.001	0.50 (0.28 to 0.86)	0.014
	Sciences	0.08 (0.03 to 0.17)	<0.001	0.89 (0.55 to 1.45)	0.644	0.83 (0.48 to 1.44)	0.508	0.37 (0.20 to 0.66)	0.001

Note: CI—Confidence Interval, AOR—Adjusted Odds Ratio.

**Table 3:** Associations between adhering to 24-h movement guidelines (HMG) and mental health N = 1469.

Mental Health Indicator	24-HMG Adherence Level (0–3)	COR (95% CI)	*p*	AOR (95% CI)	*p*
Psychological distress (high) (n = 369, 25.1%)	0	1 (Reference)		1 (Reference)	
	1	0.68 (0.41 to 1.15)	0.142	0.61 (0.36 to 1.06)	0.077
	2	0.60 (0.37 to 0.98)	0.039	0.59 (0.34 to 0.95)	0.041
	3	0.58 (0.34 to 0.98)	0.040	0.55 (0.33 to 0.95)	0.028
Low self-esteem (n = 439, 29.9%)	0	1 (Reference)		1 (Reference)	
	1	0.67 (0.41 to 1.08)	0.097	0.67 (0.39 to 1.14)	0.136
	2	0.48 (0.30 to 0.76)	0.001	0.57 (0.34 to 0.96)	0.032
	3	0.31 (0.19 to 0.52)	<0.001	0.40 (0.23 to 0.71)	0.001
Low resilience (n = 370, 25.2%)	0	1 (Reference)		1 (Reference)	
	1	0.85 (0.52 to 1.42)	0.527	0.80 (0.47 to 1.36)	0.400
	2	0.55 (0.34 to 0.90)	0.015	0.54 (0.32 to 0.92)	0.021
	3	0.37 (0.22 to 0.65)	<0.001	0.41 (0.23 to 0.74)	0.002

Note: CI—Confidence Interval, COR—Crude Odds Ratio, AOR—Adjusted Odds Ratio.

## Data Availability

De-identified data are available from the corresponding author upon reasonable request.

## Abbreviations

The following abbreviations are used in this manuscript:

24-HMG	24-h movement guidelines
PA	Physical activity
SES	Socioeconomic status
